# Polyion Complex Vesicles with Solvated Phosphobetaine Shells Formed from Oppositely Charged Diblock Copolymers

**DOI:** 10.3390/polym9020049

**Published:** 2017-02-04

**Authors:** Keita Nakai, Kazuhiko Ishihara, Michael Kappl, Syuji Fujii, Yoshinobu Nakamura, Shin-ichi Yusa

**Affiliations:** 1 Department of Applied Chemistry, University of Hyogo, 2167 Shosha, Himeji, Hyogo 671-2280, Japan; good.west.56@hotmail.co.jp; 2 Department of Materials Engineering, The University of Tokyo, 7-3-1 Hongo, Bunkyo-ku, Tokyo 113-8656, Japan; ishihara@mpc.t.u-tokyo.ac.jp; 3 Max Planck Institute for Polymer Research, Ackermannweg 10, 55128 Mainz, Germany; kappl@mpip-mainz.mpg.de; 4 Department of Applied Chemistry, Faculty of Engineering, Osaka Institute of Technology, 5-16-1 Omiya, Asahi-ku, Osaka 535-8585, Japan; syuji.fujii@oit.ac.jp (S.F.); yoshinobu.nakamura@oit.ac.jp (Y.N.)

**Keywords:** block copolymers, polyion complex, RAFT polymerization, water-soluble polymers, vesicle

## Abstract

Diblock copolymers consisting of a hydrophilic poly(2-(methacryloyloxy)ethyl phosphorylcholine) (PMPC) block and either a cationic or anionic block were prepared from (3-(methacrylamido)propyl)trimethylammonium chloride (MAPTAC) or sodium 2-(acrylamido)-2-methylpropanesulfonate (AMPS). Polymers were synthesized via reversible addition-fragmentation chain transfer (RAFT) radical polymerization using a PMPC macro-chain transfer agent. The degree of polymerization for PMPC, cationic PMAPTAC, and anionic PAMPS blocks was 20, 190, and 196, respectively. Combining two solutions of oppositely charged diblock copolymers, PMPC-*b*-PMAPTAC and PMPC-*b*-PAMPS, led to the spontaneous formation of polyion complex vesicles (PICsomes). The PICsomes were characterized using ^1^H NMR, static abd dynamic light scattering, transmittance electron microscopy (TEM), and atomic force microscopy. Maximum hydrodynamic radius (*R*_h_) for the PICsome was observed at a neutral charge balance of the cationic and anionic diblock copolymers. The *R*_h_ value and aggregation number (*N*_agg_) of PICsomes in 0.1 M NaCl was 78.0 nm and 7770, respectively. A spherical hollow vesicle structure was observed in TEM images. The hydrodynamic size of the PICsomes increased with concentration of the diblock copolymer solutions before mixing. Thus, the size of the PICsomes can be controlled by selecting an appropriate preparation method.

## 1. Introduction

Polymer vesicles prepared by self-association of block copolymers, which are of great interest because of their potential application in fields such as materials science and biochemistry. Usually, polymer vesicles are prepared by self-assembly of amphiphilic block copolymers by the solvent switch method [1,2] or the organic-solvent free method [3]. For the solvent switch method, an amphiphilic diblock copolymer is dissolved in an organic solvent that can be mixed with water, such as dimethylsulfoxide (DMSO), *N,N*-diethylformamide (DMF), tetrahydrofuran (THF), or 1,4-dioxane, to prepare a homogeneous polymer solution, followed by the gradual addition water to the organic solvent solution. The hydrophilic block chains become solvated to form the vesicle shell, which stabilizes the polymer vesicle, whereas the hydrophobic blocks associate to form the vesicle membrane. It is difficult to control vesicle size using this method because the self-assembly process depends heavily on the rate of solvent mixing. Moreover, further purification using dialysis is required to remove the organic solvent. This process is time-consuming and economically unfavorable. Furthermore, many factors, such as the initial polymer concentration, organic solvent characteristics, additives used, and temperature, affect the morphology of the polymer aggregates [4]. Polymer vesicle preparation without the use of an organic solvent usually involves rehydration of the block copolymer in water. The diblock copolymer is dissolved directly in water to form the polymer vesicle, but long and vigorous agitation is usually necessary to fully hydrate the block copolymer. However, this method results in broad size distributions [5,6].

Kataoka and Kishimura [7] reported the elegant formation of polyion complex vesicles (PICsomes) from oppositely charged diblock copolymers of cationic poly(ethylene glycol) (PEG)-*block*-poly((5-aminopentyl)-α,β-aspartamide) (PEG-P(Asp-AP)) and anionic PEG-*block*-poly(α,β-aspartic acid) (PEG-PAsp). The degree of polymerization (DP) for PEG, P(Asp-AP), and PAsp is 45, 75, and 75, respectively. Aqueous solutions of PEG-P(Asp-AP) and PEG-PAsp are prepared separately, and are mixed to prepare the PICsomes. In general, PEG is used as a hydrophilic segment for biocompatible materials because PEG suppresses non-specific protein adsorption due to its solvophilicity, large exclusion volume effect, and high mobility. The PICsome composed of PEG-P(Asp-AP) and PEG-PAsp is a potential candidate as a carrier for drug delivery systems (DDS) because the PICsome surface is surrounded by biocompatible PEG shells. However, the preparation of PICsomes involves the time-consuming processes of protection and deprotection during the syntheses of P(Asp-AP) and PAsp. Stuart and co-workers [8,9] reported water-soluble polyion complex micelles formed from oppositely charged polymers. Schrage and co-workers [10] reported PICsomes with a corona of segregated polymer chains formed from oppositely charged block ionomers in THF.

Preparation of a pair of oppositely charged doubly hydrophilic diblock copolymers, PEG-*block*-poly((3-(methacrylamido)propyl)trimethylammonium chloride) (PEG-*b*-PMAPTAC) and PEG-*block*-poly(sodium 2-(acrylamido)-2-methylpropanesulfonate) (PEG-*b*-PAMPS), was reported via reversible addition-fragmentation chain transfer (RAFT) radical polymerization using a PEG-based chain transfer agent (CTA) [11]. A stoichiometrically charged neutral mixture of these oppositely charged diblock copolymers forms water-soluble PIC micelles in water. In addition, preparation of poly(2-(methacryloyloxy)ethyl phosphorylcholine)-*block*-PMAPTAC (PMPC-*b*-PMAPTAC) and PMPC-*b*-PAMPS has been reported via RAFT using PMPC-based CTA with DP = 100 [12]. Diblock copolymers with different, well-controlled PMAPTAC (DP = 27, 48, and 96) and PAMPS (DP = 27, 45, and 99) block lengths were obtained. The PMPC possesses excellent blood compatibility, that is, PMPC does not induce hemolysis and activation of platelets when it is in contact with blood, due to a polyampholyte containing both positive and negative charges in its phosphorylcholine group [13]. Mixing aqueous solutions of PMPC-*b*-PMAPTAC and PMPC-*b*-PAMPS leads to the spontaneous formation of simple core-shell PIC micelles composed of a PIC core and PMPC shells.

**Figure 1:**
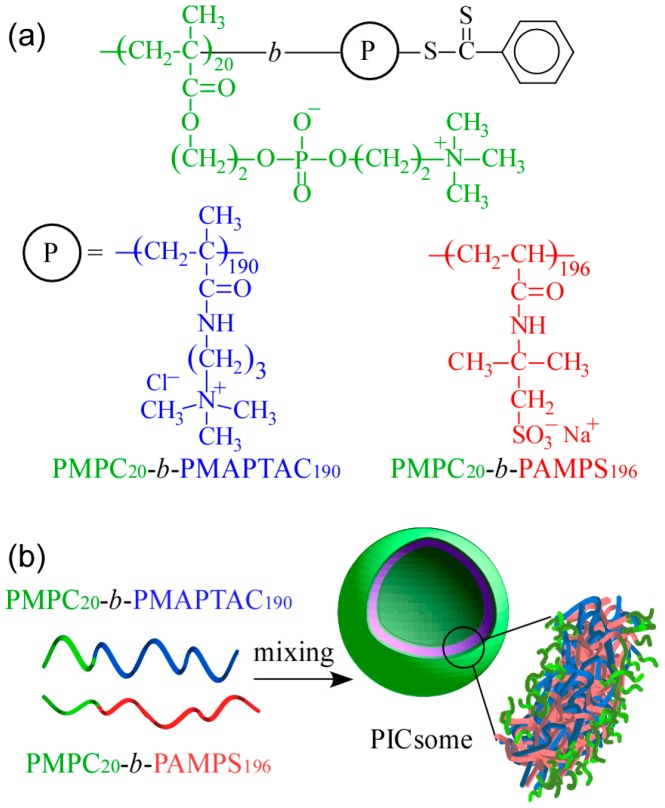
(**a**) Chemical structures of oppositely charged diblock copolymers, poly(2-(methacryloyloxy)ethyl phosphorylcholine)-*block*-poly((3-(methacrylamido)propyl) trimethylammonium chloride) (PMPC_20_-*b*-PMAPTAC_190_, P_20_M_190_) and poly(2-(methacryloyloxy) ethyl phosphorylcholine)-*block*-poly(sodium 2-(acrylamido)-2-methylpropanesulfonate) (PMPC_20_-*b*-PAMPS_196_, P_20_A_196_); (**b**) conceptual illustration of a polyion complex vesicle (PICsome) composed of a stoichiometric charge-neutral mixture of P_20_M_190_ and P_20_A_196_.

The shapes of self-assemblies formed from amphiphilic block copolymers in water are influenced by the hydrophilic/hydrophobic balance. They change from spherical micelles, thread-like micelles, and vesicles upon an increase in molecular weight of the hydrophobic block [14,15]. We would like to confirm PIC aggregates with a small volume fraction of water-soluble part form vesicle structures similar to conventional amphiphilic block copolymers. In the present study, cationic MAPTAC and anionic AMPS were polymerized using short-chain-length PMPC-based CTA with DP = 20 via RAFT radical polymerization to obtain a pair of oppositely charged diblock copolymers. These diblock copolymers were composed of short-chain-length PMPC blocks and long-chain-length charged blocks (PAMPS or PMAPTAC). The DP value of the charged blocks was about 10 times larger than that of the PMPC block. Polyion complex vesicles (PICsomes) were formed by mixing these two oppositely charged diblock copolymers, which were characterized. The expected PICsome structure is shown in Figure 1. The hydrated PMPC shells were covered on the inside and outside by a PICsome membrane composed of PMAPTAC and PAMPS blocks. This PICsome was thought to have the ability to incorporate nonionic water-soluble guest molecules into its hollow core.

## 2. Materials and Methods

### 2.1. Materials

2-(Methacryloyloxy)ethyl phosphorylcholine (MPC) was synthesized as previously reported and recrystallized from acetonitrile [16]. 4-Cyanopentanoic acid dithiobenzoate (CPD) was synthesized according to the method reported by McCormick and co-workers [17]. Methanol was dried over 4 Å molecular sieves and then distilled. The phosphate buffered saline (PBS) tablet was purchased from Sigma Aldrich (St Louis, MO, USA) and one tablet was dissolved in 200 mL purified water. (3-(Methacrylamido)propyl)trimethylammonium chloride (MAPTAC, 96%), 2-(acrylamido)-2-methylpropanesulfonic acid (AMPS, 95%), and 4,4′-azobis(4-cyanopentanoic acid) (V-501, 98%) were purchased from Wako Pure Chemical (Osaka, Japan) and Texas red-labeled dextran (Dex, *M*_w_ = 70,000, neutral) from Life Technologies (Tokyo, Japan) and were used as received without further purification. Water was purified using ion exchange. Other reagents were used as received.

### 2.2. Preparation of PMPC

The PMPC macro-chain transfer agent (PMPC macro-CTA) was prepared according to a method modified from previously reports [18]. MPC (6.03 g, 20.4 mmol) was dissolved in a mixture of methanol and water (38.8 mL, 7/5, *v*/*v*), followed by addition of CPD (423 mg, 1.38 mmol) and V-501 (48.0 mg, 0.171 mmol) to the solution. The solution was degassed by purging with argon gas for 0.5 h. Polymerization was performed at 70 °C for 6 h. The reaction mixture was dialyzed against pure water for two days. PMPC was obtained by freeze-drying (6.05 g, 93.8%). The number-average molecular weight (*M*_n_(NMR)), degree of polymerization (DP) estimated from ^1^H NMR, and molecular weight distribution (*M*_w_/*M*_n_) estimated from gel-permeation chromatography (GPC) were 6.21 × 10^3^ g/mol, 20, and 1.03, respectively.

### 2.3. Preparation of PMPC_20_-b-PMAPTAC_190_ (P_20_M_190_)

PMPC macro-CTA (345 mg, 56.5 μmol, *M*_n_(NMR) = 6.21 × 10^3^ g/mol, *M*_w_/*M*_n_ = 1.03), MAPTAC (2.50 g, 11.3 mmol), and V-501 (7.90 mg, 28.2 μmol) were dissolved in water (22.6 mL). The solution was deoxygenated by purging with argon gas for 0.5 h. Polymerization was conducted at 70 °C for 6 h. The diblock copolymer was purified by dialysis against pure water for two days. The cationic diblock copolymer (P_20_M_190_) was recovered using freeze-drying (2.46 g, 85.9%, *M*_n_(NMR) = 4.95 × 10^4^ g/mol, *M*_w_/*M*_n_ = 1.05).

### 2.4. Preparation of PMPC_20_-b-PAMPS_196_ (P_20_A_196_)

A predetermined amount of AMPS (2.00 g, 9.67 mmol) was neutralized with 1 M NaOH in 9.65 mL of water. Then, PMPC macro-CTA (300 mg, 48.3 μmol, *M*_n_(NMR) = 6.21 × 10^3^ g/mol, *M*_w_/*M*_n_ = 1.03) and V-501 (10.7 mg, 38.2 μmol) was added to the solution, which was deoxygenated by purging with argon gas for 30 min. Polymerization was performed at 70 °C for 3 h. The diblock copolymer was purified by dialysis against pure water for two days. The anionic diblock copolymer (P_20_A_196_) was obtained by freeze-drying (2.25 g, 88.8%, *M*_n_(NMR) = 4.85 × 10^4^ g/mol, *M*_w_/*M*_n_ = 1.07).

### 2.5. Preparation of Polyion Complex Vesicles (PICsomes)

The P_20_M_190_ and P_20_A_196_ were dissolved separately in NaCl aqueous solutions, and the solutions were left standing overnight at room temperature to achieve complete dissolution. A P_20_M_190_ solution was added dropwise to a P_20_A_196_ solution over a period of 5 min at room temperature with stirring to prepare the PIC vesicles (PICsomes), and the mixture was left standing for at least 1 h prior to measurement. The mixing ratio of the block copolymers was represented by the mole fraction of positively charged unit (*f*^+^ = [MAPTAC]/([AMPS] + [MAPTAC])) and hence complete charge neutralization was achieved at *f*^+^ = 0.5.

### 2.6. Encapsulation of Texas Red-Labeled Dextran (Dex)

Dex (0.040 mg, 5.71 × 10^−10^ mol) was dissolved in PBS buffer (4 mL), and P_20_M_190_ (0.5 g/L) and P_20_A_196_ (0.5 g/L) were dissolved in PBS buffer solutions containing Dex separately. The solutions were allowed to stand overnight at room temperature. The P_20_M_190_ solution was added to the P_20_A_196_ solution over a period of 5 min with stirring. The *f*^+^ value was kept constant at 0.5. The solution (4 mL) was dialyzed using a polycarbonate membrane with 100-nm pore size (Harvard Apparatus, Holliston, MA, USA) against fresh PBS buffer (400 mL) for 18 h, changing the PBS buffer 3 times to remove the free Dex that was not incorporated into the hollow core of the PICsome. After dialysis, fluorescence emission of the PBS buffer in the dialyzer was measured. As a reference, fluorescence of the PBS buffer solution of Dex without PICsomes was also measured using a similar procedure. The weight of the Dex incorporated into the PICsomes was calculated using a calibration curve. The loading efficiency (LE) and loading capacity (LC) of Dex were calculated according to the following equations:

(1) $$\mathrm{LE}\;(\%)=\frac{\mathrm{Weight}\;\mathrm{of}\;\mathrm{encapsulated}\;\mathrm{Dex}}{\mathrm{Weight}\;\mathrm{of}\;\mathrm{total}\;\mathrm{Dex}}\times 100,$$

(2) $$\mathrm{LC}\;(\%)=\frac{\mathrm{Weight}\;\mathrm{of}\;\mathrm{encapsulated}\;\mathrm{Dex}}{\mathrm{Weight}\;\mathrm{of}\;\mathrm{polymer}}\times 100.$$

### 2.7. Measurements

The GPC measurements for the cationic polymer were obtained using a Jasco (Tokyo, Japan) RI-2031 Plus refractive index detector equipped with a Jasco PU-8020 pump and a Shodex (Tokyo, Japan) OHpak SB-804 HQ column (exclusion limit ~10^7^) working at 40 °C under a flow rate of 0.60 mL/min. A 0.30 M aq. Na_2_SO_4_ solution containing 0.50 M acetic acid was used as the eluent. The values of *M*_n_(GPC) and *M*_w_/*M*_n_ were calibrated using standard poly(2-vyniypyridine) samples. The GPC measurements for the anionic polymer were obtained using a Tosoh RI-8020 refractive index detector (Tosoh, Tokyo, Japan) equipped with a Shodex 7.0-μm bead size GF-7M HQ column (exclusion limit ~10^7^) working at 40 °C under a flow rate of 0.60 mL/min. A phosphate buffer (50 mM, pH 9.0) containing 10 vol % acetonitrile was used as the eluent. The values of *M*_n_(GPC) and *M*_w_/*M*_n_ were determined using standard sodium poly(styrenesulfonate) samples. ^1^H NMR spectra were obtained with a Bruker (Yokohma, Japan) DRX-500 spectrometer operating at 500.13 MHz with a deuterium lock. Light-scattering measurements were performed using an Otsuka Electronics Photal (Osaka, Japan) DLS-7000HL equipped with a multi-τ, digital time correlator (ALV-5000E). A helium-neon (He-Ne) laser (10.0 mW at 632.8 nm) was used as a light source. Sample solutions for light scattering measurements were filtered with a 0.45-μm membrane filter. From static light scattering (SLS) measurements, the weight-average molecular weight (*M*_w_), *z*-average radius of gyration (*R*_g_), and second virial coefficient (*A*_2_) values were calculated by the relation:

(3) $$\frac{KC_{p}}{R_{\theta }}=\frac{1}{M_{w}}\left(1+\frac{1}{3}{R_{g}}^{2}q^{2}\right)+2A_{2}C_{p},$$

where *R*_θ_ is the difference between the Rayleigh ratio of the solution and that of the solvent, *K* = 4π^2^*n*^2^(d*n*/d*C*_p_)^2^/*N*_A_λ^4^ with d*n*/d*C*_p_ being the refractive index increment against *C*_p_, *N*_A_ being Avogadro’s number, and *q* the magnitude of the scattering vector. The *q* value was calculated from *q* = (4π*n*/λ)sin(θ/2), where *n* is the refractive index of the solvent, λ is the light source wavelength (=632.8 nm), and θ is the scattering angle. By measuring *R*_θ_ for a set of *C*_p_ and θ, values of *M*_w_, *R*_g_, and *A*_2_ were estimated from Zimm plots. The known Rayleigh ratio of toluene was used for calibration of the instrument. Values of d*n*/d*C*_p_ at 633 nm were determined using an Otsuka Electronics Photal (Osaka, Japan) DRM-3000 differential refractometer. In our dynamic light scattering (DLS) experiments, inverse Laplace transform (ILT) analysis was performed using the REPES algorithm [19,20,21] to obtain the relaxation time distribution, τ*A*(τ). The relaxation rate (Γ = τ^−1^) is a function of θ [22]. The diffusion coefficient in the limit of zero angle (*D*) was calculated from *D* = (Γ/*q*^2^)*_q_*_→0_. The hydrodynamic radius (*R*_h_) was provided by the Stokes–Einstein equation, *R*_h_ = *k*_B_*T*/(6πη*D*), where *k*_B_ is Boltzmann constant, *T* is absolute temperature, and η is solvent viscosity. The ζ-potential measurements were obtained using a Malvern (Worcestershire, UK) Zetasizer Nano-ZS ZEN3600 equipped with a He–Ne laser light source (4 mW at 632.8 nm). The ζ-potential was calculated from the electrophoretic mobility (μ) using the Smoluchowski relation, ζ = ημ/ε (κ*a* >> 1), where η is viscosity, ε is the dielectric constant of the medium, and κ and *a* are the Debye–Hückel parameter and particle radius, respectively [23]. Transmission electron microscopy (TEM) observations were performed using a Jeol JEM-2100 instrument at an accelerating voltage of 200 kV. Samples for TEM were prepared by placing one drop of the aqueous solution on a copper grid coated with thin films of Formvar. Excess water was blotted using filter paper. The samples were stained by sodium phosphotungstate and dried under vacuum for one day. Atomic force microscope (AFM) observations were performed with a JPK Nano Wizard 3 (JPK Instruments, Berlin, Germany) microscope. The sample of PICsome was applied onto a freshly cleaved mica surface. Excess water was blotted using filter paper and the sample dried for 10 min at 25 °C. Measurements were obtained in tapping mode using the Olympus (Tokyo, Japan) OMCLAC 160 TN-W2 silicon AFM probes (nominal spring constant, *k* = 42 N/m, resonance frequency ca. 300 kHz, tip radius < 10 nm). Height and size information were extracted using JPK data processing software (Version 5.1.8, JPK Instruments, Berlin, Germany). Fluorescence emission spectra were recorded on a Hitachi (Tokyo, Japan) F-2500 fluorescence spectrophotometer. Fluorescence spectra of Dex were measured with excitation at 550 nm. Excitation and emission slit widths were maintained at 10 nm.

## 3. Results and Discussion

To obtain oppositely charged diblock copolymers (P_20_M_190_ and P_20_A_196_), block copolymerization was conducted using PMPC macro-CTA with DP = 20 via RAFT radical polymerization. The conversions of MAPTAC and AMPS were estimated from ^1^H NMR measurements after polymerization reached 93.0% and 95.0%, respectively. The molecular characteristics of PMPC, P_20_M_190_, and P_20_A_196_ are summarized in Table 1. The theoretical number-average molecular weight (*M*_n_(theo)) was calculated using:

(4) $$M_{n}\left(theo\right)=\frac{{\left[M\right]}_{0}}{{\left[CTA\right]}_{0}}x_{m}M_{m}+M_{CTA},$$

where [M]_0_ is initial monomer concentration, [CTA]_0_ is initial CTA concentration, *x*_m_ is the conversion of monomer, *M*_m_ is the molecular weight of the monomer, and *M*_CTA_ is the molecular weight of the CTA. The *M*_n_(NMR) and *M*_n_(GPC) for PMPC were close to the theoretical *M*_n_(theo) value, and the molecular weight distribution (*M*_w_/*M*_n_) was narrow (=1.03), indicating the controlled mechanism of the polymerization. Values obtained from ^1^H NMR to determine the true molecular weight of P_20_M_190_ and P_20_A_196_ yielded *M*_n_(NMR) = 4.95 × 10^4^ and 4.85 × 10^4^ g/mol, which was in fair agreement with *M*_n_(theo) = 4.82 × 10^4^ and 4.68 × 10^4^ g/mol, respectively. The *M*_n_(GPC) value for P_20_M_190_ and P_20_A_196_ deviated markedly from *M*_n_(theo). Note that the *M*_n_(GPC) values estimated by GPC were only apparent values because of the inherent error involved in the use of molecular weight standards [poly(2-vinylpyridine) and sodium poly(styrenesulfonate)] for calibrating the GPC data.

**Figure 2:**
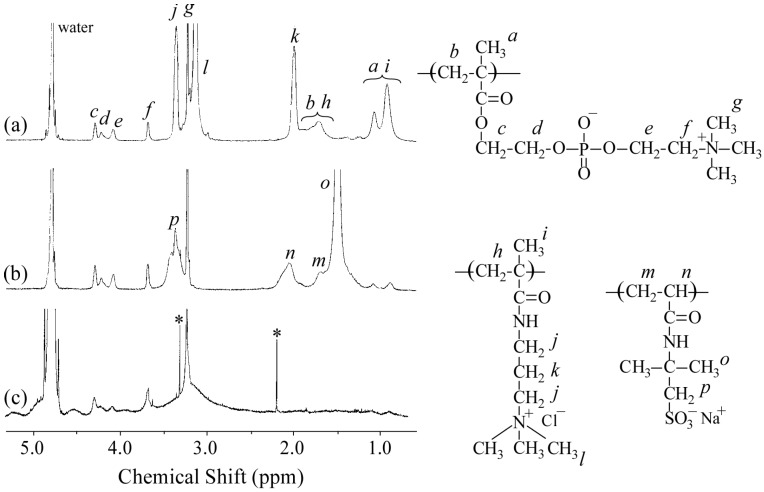
^1^H NMR spectra for (**a**) P_20_M_190_; (**b**) P_20_A_196_; and (**c**) PICsome composed of P_20_M_190_ and P_20_A_196_ with *f*^+^ = 0.5 in a D_2_O solution containing 0.1 M NaCl. Resonance peak assignments are indicated.

Figure 2a,b show the ^1^H NMR spectra for P_20_M_190_ and P_20_A_196_, respectively. The resonance bands observed in the 0.8–1.2 ppm region and at 1.8 ppm were attributed to the α-methyl protons and main chain methylene protons, respectively (Figure 2a). The DP and *M*_n_(NMR) values of the PMAPTAC block in P_20_M_190_ were determined from the integral intensity ratio of the resonance bands due to pendant methyl protons in the PMAPTAC block at 3.1 ppm and PMPC pendant methylene protons at 3.7 ppm. The resonance bands observed at 1.2–2.2 ppm for P_20_A_196_ were attributed to the sum of the main chain and pendent methyl groups in the PAMPS block (Figure 2b). Values for DP and *M*_n_(NMR) of the PAMPS block in P_20_A_196_ were calculated from the integral intensity ratio of the pendent methylene protons in the PAMPS block at 3.3 ppm and PMPC pendant methylene protons at 3.7 ppm. Figure 2c shows the ^1^H NMR spectrum for the polyion complex vesicle (PICsome) composed of P_20_M_190_ and P_20_A_196_ with *f*^+^ = 0.5 in D_2_O containing 0.1 M NaCl. The intensity of resonance peaks associated with the PMAPTAC and PAMPS blocks was weak compared with those associated with the PMPC block. These observations suggest that the motion of the PMAPTAC and PAMPS blocks was highly restricted due to formation of the PIC core. The mobility of PMPC chains may be higher than that of the PMAPTAC and PAMPS chains because the PMPC chains form shells surrounding the PIC.

Aggregates formed by electrostatic interactions sometimes depend on the mixing pathway [24,25,26]. We studied PICsome size dependence on the mixing pathway. A standard method is that a P_20_M_190_ solution was added dropwise to a P_20_A_196_ solution over a period of 5 min at room temperature with stirring. The P_20_A_196_ solution was added to the P_20_M_190_ solution, and the P_20_M_190_ solution was added to the P_20_A_196_ solution immediately. These two additional methods had no effect on the size of PICsome with *f*^+^ = 0.5.

**Figure 3:**
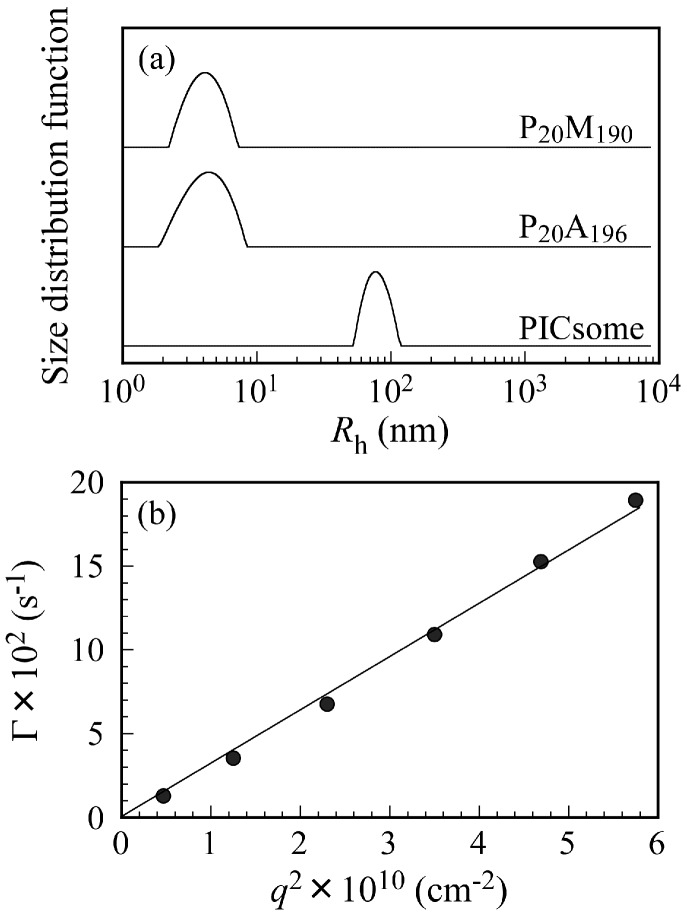
(**a**) Hydrodynamic radius (*R*_h_) distributions for P_20_M_190_, P_20_A_196_, and PICsome with *f*^+^ = 0.5 in aq. 0.1 M NaCl at *C*_p_ = 0.5 g/L and at scattering angle (θ) = 90°; (**b**) relation between relaxation rate (Γ) and square of the magnitude of the scattering vector (*q*^2^) for PICsome with *f*^+^ = 0.5 at *C*_p_ = 0.5 g/L.

Figure 3a shows *R*_h_ distributions for P_20_M_190_, P_20_A_196_, and the PICsome with *f*^+^ = 0.5 in 0.1 M NaCl at *C*_p_ = 0.5 g/L and a scattering angle (θ) = 90°. Unimodal *R*_h_ distributions were observed. The *R*_h_ values for P_20_M_190_, P_20_A_196_, and PICsome were 4.3, 4.4, and 78.0 nm, respectively. The *R*_h_ values from 4.3 to 4.4 nm are reasonable for unimers of these block copolymers. If the polymer main chain forms completely planar zigzag structure, the distance between one carbon to the next carbon is about 0.25 nm [27]. Hence, we can calculate the end-to-end distance of fully expanded polymer chains. The end-to-end distance of fully extended P_20_M_190_ and P_20_A_196_ chains were calculated as 52.5 and 54.0 nm, respectively. The *R*_h_ of 78.0 nm found for the PICsome was larger than those expected from the fully extended length of the P_20_M_190_ and P_20_A_196_ chains. These observations indicate that the shape of the PICsome is not a simple core-shell spherical micelle. Large compound aggregates or vesicles should be formed by mixing P_20_M_190_ and P_20_A_196_. Relaxation rates (Γ) measured at different θ plotted against the square of the scattering vector (*q*^2^) are shown in Figure 3b. A line passing through the origin suggests that all of the relaxation modes were virtually diffusive [28].

To confirm the stability of the PICsome size, *R*_h_ values were measured at various standing times. The *R*_h_ values were nearly constant and independent of time until 150 h, suggesting that the structure of the PICsome does not change with time (data not shown). Scattering intensities of the PICsomes were also independent of time.

To further characterize the PICsomes, SLS measurements were performed for θ from 30 to 130° with a 20° increment. The refractive index increment (d*n*/d*C*_p_) for P_20_M_190_, P_20_A_196_, and PICsome in 0.1 M NaCl were determined individually. Values for *M*_w_(SLS), *R*_g_, and *A*_2_ were estimated from Zimm plots. Aggregation number (*N*_agg_) for PICsomes (i.e., number of PMPC shell chains per one PICsome) was calculated by dividing *M*_w_(SLS) with that of unimers. The structure of the PICsome was also characterized by combining DLS and SLS to determine the *R*_g_/*R*_h_ ratio. The density (*d*) of P_20_M_190_, P_20_A_196_, and PICsome can be calculated by:

(5) $$d=\frac{M_{w}(\mathrm{SLS})}{N_{A}\times V},$$

where *V* is a polymer or PICsome volume calculated from 4/3π*R*_h_^3^. A summary of the properties of P_20_M_190_, P_20_A_196_, and the PICsome, including *M*_w_(SLS), *N*_agg_, *R*_g_, *R*_h_, *R*_g_/*R*_h_, *A*_2_, d*n*/d*C*_p_, and *d*, is provided in Table 2. Values for *M*_w_(SLS) for P_20_M_190_ and P_20_A_196_ were close to those for the corresponding *M*_n_(theo) and *M*_n_(NMR) values shown in Table 1. The *M*_w_(SLS) value for the PICsome was 4.50 × 10^8^ g/mol, estimated from SLS. The value of *N*_agg_ for the PICsome was estimated to be 7770. The *R*_g_/*R*_h_ ratio is a structure-sensitive parameter that provides information about the density distribution of the particles and thereby about particle morphology [29,30]. The *R*_g_/*R*_h_ ratio equals 0.775 for a homogeneous hard sphere, 1.0 for a thin hard spherical shell (e.g., vesicle), and increases significantly for a less dense structure and for a polydisperse solution because large molecules of a broad distribution will contribute more to *R*_g_ than to *R*_h_, provided that internal modes of motion are absent [31]. The large *R*_g_/*R*_h_ ratios (>4) for P_20_M_190_ and P_20_A_196_ suggest that the polymer chains were expanded due to electrostatic repulsions in the pendant ions. The *R*_g_/*R*_h_ ratio for a polymeric vesicle may be less than or greater than 1.0, depending on the thickness and density of the wall [32]. The *R*_g_/*R*_h_ ratio of the PICsome was 1.12, which is close to unity, indicates that the PICsome was a vesicle [33]. The *A*_2_ value for the PICsome was less than those for P_20_M_190_ and P_20_A_196_, suggesting that solubility of the PICsome in 0.1 M NaCl was less than those of the unimers. The *d* values for P_20_M_190_, P_20_A_196_, and PICsome were calculated to be 0.286, 0.273, and 0.376 g/cm^3^, respectively. The *d* value for the PICsome was slightly larger than those for P_20_M_190_ and P_20_A_196_, suggesting that the polymer chains in the PICsome were more densely packed than those of the unimers. The polymer chains of P_20_M_190_ and P_20_A_196_ expanded due to electrostatic repulsion in the pendant ionic groups in 0.1 M NaCl. Therefore, P_20_M_190_ and P_20_A_196_ have large *R*_g_/*R*_h_ ratios. In contrast, the polymer chains in the PICsome were compact and dense in their vesicular membranes.

**Figure 4:**
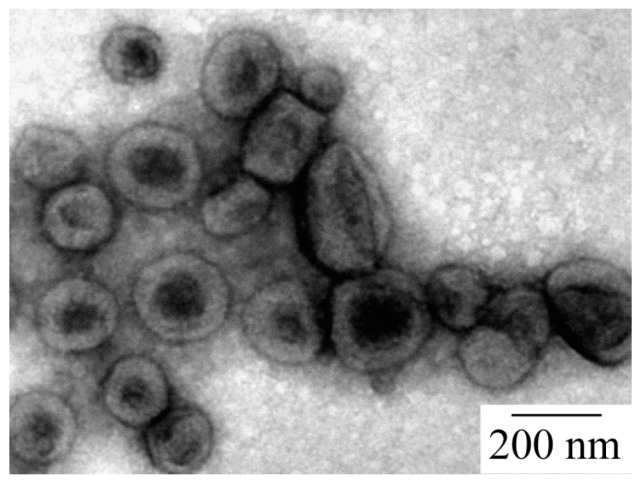
Transmission electron microscopy (TEM) image of the PICsome composed of P_20_M_190_ and P_20_A_196_ with *f*^+^ = 0.5.

**Figure 5:**
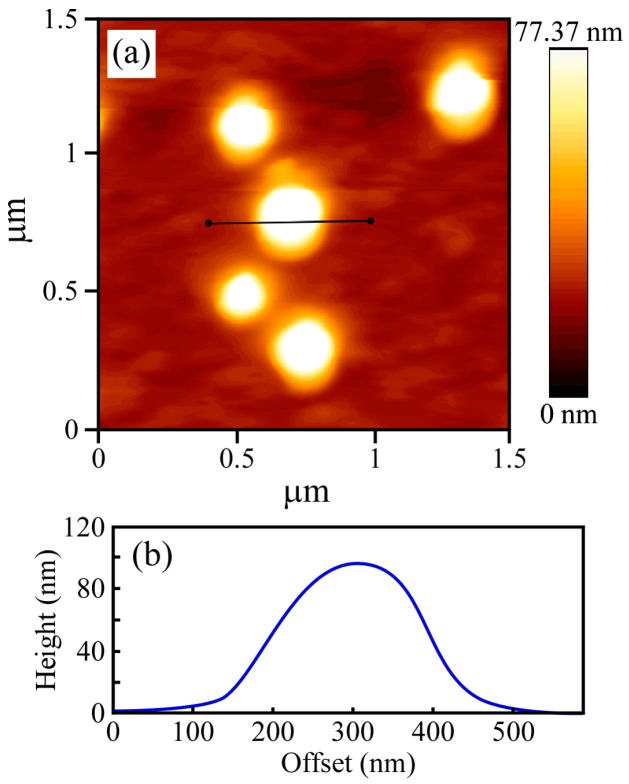
(**a**) Atomic force microscope (AFM) height image and (**b**) corresponding height cross-section for the PICsome with *f*^+^ = 0.5.

The structure of the PICsome was confirmed by TEM observations, which showed incomplete spherical hollow vesicle structures (Figure 4). The vesicle structures may shrink during the drying process done prior to TEM observation. The PICsome diameter determined from the TEM images was 171 nm, which is close to the value obtained from the light scattering data. The AFM height image of the PICsome confirmed that the PICsome formed spherical structures that were slightly flattened due to the adsorption and drying process (Figure 5). The height of the PICsome observed in the AFM image was ca. 100 nm. 

**Figure 6:**
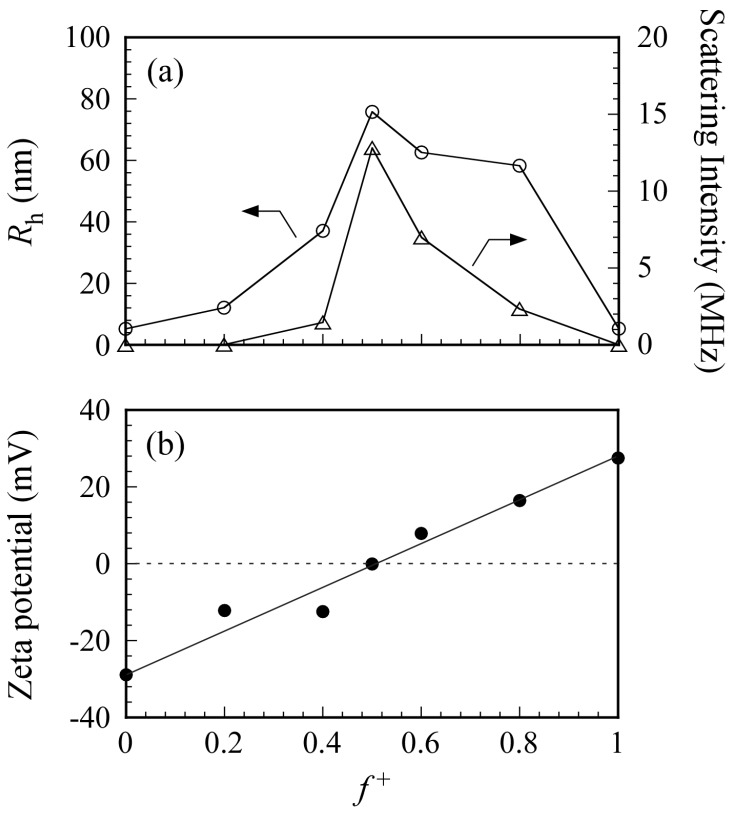
(**a**) Hydrodynamic radius (*R*_h_, ○) and photon counting rate which indicates light scattering intensity (△) for the PICsome composed of P_20_M_190_ and P_20_A_196_ as a function of *f*^+^ (= [MAPTAC]/([MAPTAC] + [AMPS])) in 0.1 M NaCl; (**b**) ζ-potential for PICsome as a function of *f*^+^ in 0.1 M NaCl. Total polymer concentration was fixed at 0.5 g/L.

**Figure 7:**
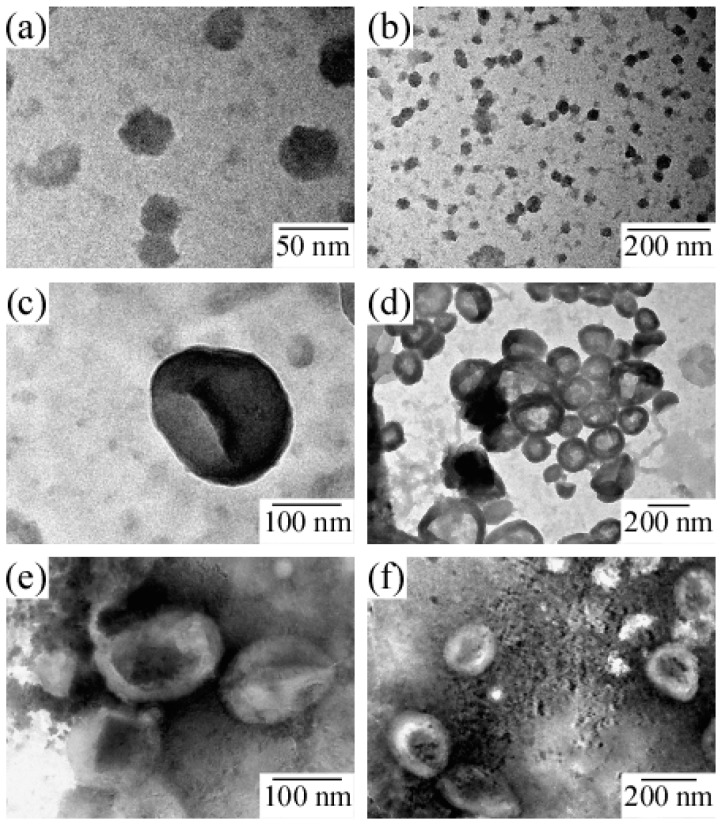
TEM images for PIC aggregates with *f*^+^ values of (**a**,**b**) 0.4, (**c**,**d**) 0.6, and (**e**,**f**) 0.8.

Figure 6a shows the *R*_h_ and light scattering intensity values for PICsomes in 0.1 M NaCl as a function of *f*^+^. Total polymer concentration was kept constant at 0.5 g/L. An increase in *R*_h_ indicates an increase in the size of the PICsome. The maximum *R*_h_ value was observed at *f*^+^ = 0.5. In general, scattering intensity depends on molecular weight of the particles. Therefore, an increase in scattering intensity indicates an increase in *N*_agg_ for the PICsome, which suggests that stoichiometric charge neutralization in the mixture of the two oppositely charged P_20_M_190_ and P_20_A_196_ leads to formation of PICsomes with maximum size and aggregation number. Plots of *R*_h_ (and scattering intensity) vs. *f*^+^ were asymmetric (i.e., the *R*_h_ and scattering intensities for PIC aggregates with *f*^+^ = 0.6 and 0.8 were larger than those with *f*^+^ = 0.4) [34]. To confirm the structure of PIC aggregates with *f*^+^ = 0.4, 0.6, and 0.8, TEM images were obtained (Figure 7). Results showed that PIC aggregates with *f*^+^ = 0.4 were micelle-like spherical particles without a hollow core. In contrast, PIC aggregates with *f*^+^ = 0.6 and 0.8 clearly possessed hollow core vesicle structures. The PIC aggregates composed of P_20_M_190_ and P_20_A_196_ with excess PMAPTAC blocks tended to form vesicles, presumably because the pendant quaternary amino groups surrounded by three methyl groups in the PMAPTAC blocks were more hydrophobic compared to the pendant sulfonate groups in the PAMPS blocks. When *f*^+^ is larger than 0.5, excess PMAPTAC blocks existed in the aggregate, dehydration of PIC aggregates was promoted, and solubility was less than that at *f*^+^ < 0.5. For aggregates formed from conventional amphiphilic diblock copolymers in water, the greater the hydrophobicity of the aggregate, the more likely diblock copolymers are to form vesicles rather than spherical core-shell micelles [35]. Therefore, PIC aggregates with *f*^+^ ≥ 0.5 tend to form vesicles.

To confirm PICsome neutralization at *f*^+^ = 0.5, the ζ-potential was measured as a function of *f*^+^ (Figure 6b). At *f*^+^ = 0, the aqueous solution of P_20_A_196_ has a negative ζ-potential value of −29 mV because the PAMPS block has pendant anionic sulfonate groups. At *f*^+^ = 1, the aqueous solution of P_20_M_190_ has a positive ζ-potential value of +27 mV because the PMAPTAC block has pendant cationic quaternary amino groups. The ζ-potential was zero at *f*^+^ = 0.5 because the charges of the PAMPS and PMAPTAC blocks were neutralized. The PICsome was composed of a PIC core and PMPC shells. The pendant phosphorylcholine groups in the PMPC shells contain anionic phosphate and cationic quaternary amine. However, the ζ-potential of PMPC homopolymer was zero (data not shown) because of neutralization of the anion and cation pair within a single polymer chain. Therefore, the ζ-potential of PICsome was zero at *f*^+^ = 0.5.

**Figure 8:**
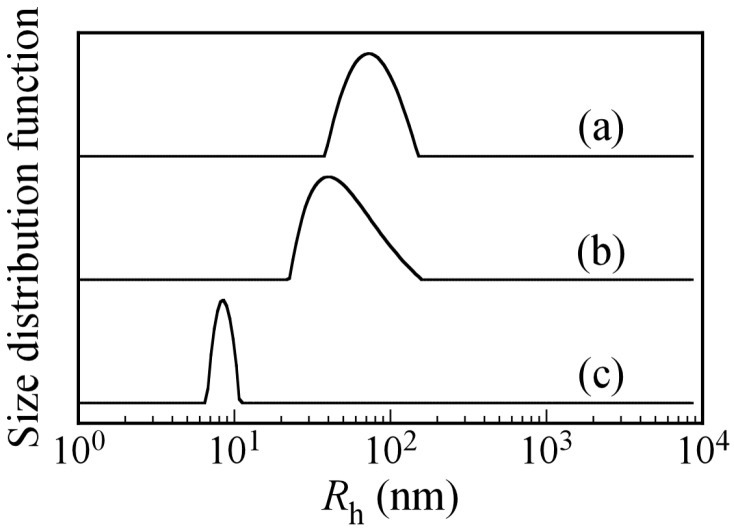
Hydrodynamic radius (*R*_h_) distributions for (**a**) PICsomes at *f*^+^ = 0.5, and PIC aggregates (**b**) at *f*^+^ = 0.4 and (**c**) *f*^+^ = 0.2 formed by addition of P_20_A_196_ solution to PICsomes with *f*^+^ = 0.5 in 0.1 M aqueous NaCl solutions at *C*_p_ = 0.5 g/L.

To confirm that PICsomes with *f*^+^ = 0.5 are at equilibrium or in a kinetically frozen state, excess P_20_A_196_ was added to the aqueous PICsome solution with *f*^+^ = 0.5 to change the *f*^+^ value. A kinetically frozen state means that the polymer chains cannot break free from the aggregate. The size of a PICsome with *f*^+^ = 0.5 in the kinetically frozen state should not be affected by the addition of excess P_20_A_196_. The size of a PICsome in the equilibrium state may decrease upon addition of excess P_20_A_196_. Figure 8 shows *R*_h_ distributions for PICsomes with *f*^+^ = 0.5 and PIC aggregates with *f*^+^ = 0.4 and 0.2 formed by the addition of P_20_A_196_ to the PICsome with *f*^+^ = 0.5. The *R*_h_ value of the PICsome with *f*^+^ = 0.5 was 78.0 nm at *C*_p_ = 0.5 g/L. When a P_20_A_196_ solution at *C*_p_ = 0.5 g/L was added to the PICsome solution, which changed the *f*^+^ to 0.4 and 0.2, the *R*_h_ values of the PIC aggregates decreased to 49 and 7.9 nm, respectively. This observation suggested that PICsomes formed by mixing oppositely charged diblock copolymers existed in an equilibrium state in water. Thus, small pairs of the oppositely charged diblock copolymers may dissociate from and associate with the PICsome [36]. NaCl concentrations in the aqueous solution are very important for stability of PICsomes because they were formed by electrostatic interactions. We measured the *R*_h_ values of PICsomes in various NaCl concentrations. When NaCl concentration was 0.5 M, the *R*_h_ value was 77.8 nm, which is close to the value (*R*_h_ = 78.0 nm) in 0.1 M NaCl aqueous solutions. Therefore, at least below 0.5 M of NaCl concentration, PICsomes were stable. 

**Figure 9:**
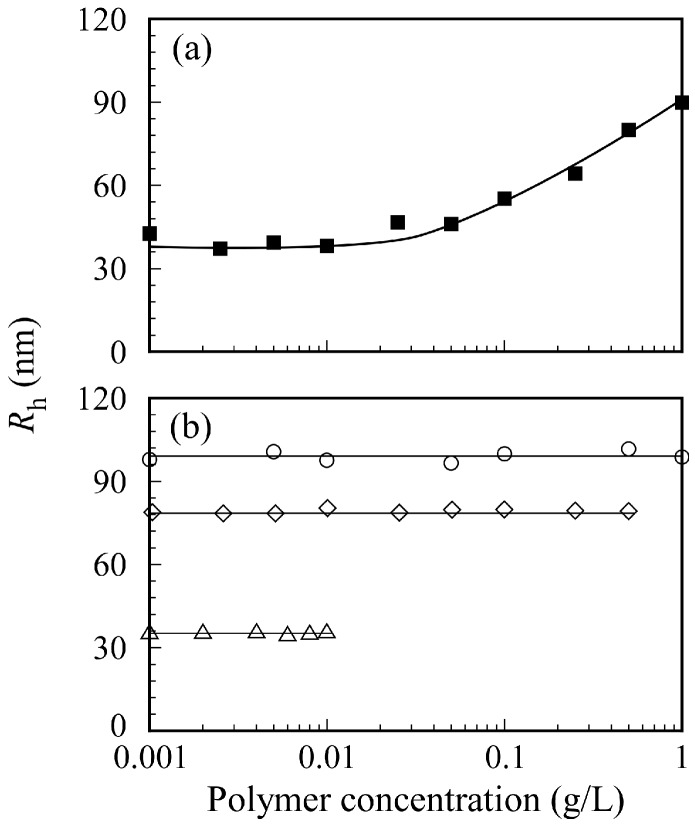
(**a**) Hydrodynamic radius (*R*_h_) for PICsomes with *f*^+^ = 0.5 as a function of polymer concentration of P_20_M_190_ and P_20_A_196_ before mixing both block copolymers in 0.1 M NaCl; (**b**) *R*_h_ for PICsomes with *f*^+^ = 0.5 as a function of polymer concentration after mixing P_20_M_190_ and P_20_A_196_. The aq. PICsome solutions at 1 (○), 0.5 (◇), and 0.01 g/L (△) were diluted with 0.1 M NaCl continuously.

The relation between PICsome size and *C*_p_ in 0.1 M NaCl is shown in Figure 9. The sample solutions were prepared by two different methods. The first method involved preparing separate aqueous P_20_M_190_ and P_20_A_196_ solutions with a target *C*_p_ from 0.001 to 1 g/L before mixing a pair of two oppositely charged diblock copolymers. Then, the two aqueous P_20_M_190_ and P_20_A_196_ solutions with the same *C*_p_ were mixed to form a PICsome solution (Figure 9a). The second method involved mixing pairs of oppositely charged diblock copolymer solutions at *C*_p_ = 1, 0.5, and 0.01 g/L to form PICsomes. Subsequently, the aqueous PICsome solutions were diluted with 0.1 M NaCl to adjust *C*_p_ to the target value (Figure 9b). These PICsome solutions prepared via these two *C*_p_ adjustment methods were measured using DLS to determine *R*_h_. The *R*_h_ values for PICsomes depended on the value of *C*_p_ of the P_20_M_190_ and P_20_A_196_ aqueous solutions before mixing to form the PICsome. When each P_20_M_190_ and P_20_A_196_ solution was prepared at *C*_p_ = 1 g/L, the *R*_h_ value for the PICsome was ca. 100 nm. In contrast, when each aqueous P_20_M_190_ and P_20_A_196_ solution was prepared at *C*_p_ = 0.01 g/L, the *R*_h_ value for the PICsome was ca. 38 nm. The size of the PICsomes could be controlled by adjusting the *C*_p_ values of oppositely charged diblock copolymer solutions before mixing. When 0.1 M NaCl PICsome solutions were diluted with 0.1 M NaCl, the *R*_h_ values for the PICsome remained nearly constant, independent of *C*_p_. These findings suggest that the structure of the PICsome, once prepared, is stable against dilution. In general, sonication and extrusion techniques are used to control the size of vesicles [37]. However, the easily adjustable size of the stable PICsome system described here indicates that the size of vesicles and polymersomes can be easily controlled by adjusting the *C*_p_ before mixing a pair of oppositely charged diblock copolymers.

**Figure 10:**
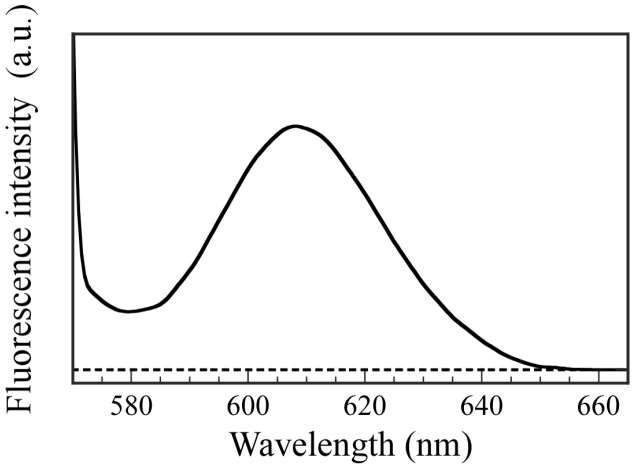
Fluorescence emission spectra for Dex after dialysis against PBS buffer for 18 h excited at 550 nm in the presence (──) and absence (----) of PICsomes in PBS buffer solutions.

To confirm the ability to incorporate hydrophilic guest molecules into the interior aqueous phase of PICsomes, fluorescence experiments were performed using Texas red-labeled Dex as a fluorescence probe. The hydrophilic Dex molecule contains no charged groups. The P_20_M_190_ and P_20_A_196_ were dissolved in Dex-containing PBS buffer solutions, and then these solutions were mixed to form PICsomes. The Dex molecules that could not be incorporated into the PICsomes were removed by dialysis against fresh PBS buffer for 18 h. Fluorescence spectra were obtained for the solution inside the dialyzer after dialysis (Figure 10). Fluorescence emission with a maximum wavelength at 610 nm for Dex was observed, which indicates that the Dex molecules were incorporated into the hollow core of PICsome. In contrast, a blank solution in the absence of PICsomes produced no fluorescence from Dex because the small Dex molecules were removed when using a dialysis membrane with a pore size of 100 nm. These results demonstrate that PICsomes can incorporate Dex guest molecules into the hollow core. The weight of the Dex incorporated into the PICsomes was calculated using a calibration curve, and was 0.00315 mg. The LE and LC values determined using the encapsulated Dex weight were 78.8% and 1.58%, respectively. 

## 4. Conclusions

A pair of oppositely charged diblock copolymers with well-controlled structures, P_20_M_190_ and P_20_A_196_, were prepared via RAFT using PMPC macro-CTA. Polyion complex vesicles (PICsomes) were formed by stoichiometric charge neutralization of a mixture of aqueous P_20_M_190_ and P_20_A_196_ solutions. The surface of the PICsomes was covered with biocompatible PMPC shell chains. These PICsomes could incorporate water-soluble guest molecules without charge groups inside the interior aqueous phase, which indicates that these PICsomes may be useful as a molecular carrier of several bioactive compounds.

## Figures and Tables

**Table 1:** Number-average molecular weight (*M*_n_), number-average degree of polymerization (DP), and molecular weight distribution (*M*_w_/*M*_n_) for the samples.

Sample	*M*_n_(theo) *^a^* × 10^4^ (g/mol)	*M*_n_(NMR) × 10^4^ (g/mol)	DP(NMR)	*M*_n_(GPC) × 10^4^ (g/mol)	*M*_w_/*M*_n_
PMPC	0.613	0.621	20	0.735 *^b^*	1.03 *^b^*
P_20_M_190_	4.82	4.95	190	2.40 *^c^*	1.05 *^c^*
P_20_A_196_	4.68	4.85	196	2.77 *^b^*	1.07 *^b^*

*^a^* Calculated using Equation (4). *^b^* Estimated from gel-permeation chromatography (GPC) using phosphate buffer (50 mM, pH 9.0) containing 10 vol % acetonitrile as eluent. *^c^* Estimated from GPC using aq. 0.30 M Na_2_SO_4_ containing 0.50 M acetic acid as eluent.

**Table 2:** Dynamic and static light scattering data for P_20_M_190_, P_20_A_196_, and polyion complex vesicles (PICsomes).

Sample	*M*_w_(SLS) *^a^* × 10^4^ (g/mol)	*N*_agg_ *^b^*	*R*_g_ *^c^* (nm)	*R*_h_ *^d^* (nm)	*R*_g_/*R*_h_	*A*_2_ *^e^* (cm^3^mol/g^2^) × 10^−4^	d*n*/d*C*_p_ *^f^* (mL/g)	*d ^g^* (g/cm^3^)
P_20_M_190_	5.73	1	22.2	4.3	5.14	8.13	0.166	0.286
P_20_A_196_	5.86	1	21.1	4.4	4.83	12.4	0.141	0.273
PICsome *^h^*	45,000	7770	87.0	78.0	1.12	2.34	0.161	0.376

*^a^* Apparent weight-average molecular weight estimated from static light scattering (SLS). *^b^* Aggregation number of PICsomes calculated by dividing *M*_w_(SLS) with that of unimers. *^c^* Radius of gyration estimated from SLS. *^d^* Hydrodynamic radius estimated from dynamic light scattering (DLS). *^e^* Second virial coefficient estimated from SLS. *^f^* Refractive index increment. *^g^* Density of polymers and PICsomes calculated from Equation (5). *^h^* PICsome composed of P_20_M_190_ and P_20_A_196_ with *f*^+^ = 0.5.
